# Address

**Published:** 1866-09

**Authors:** I. J. Wetherbee


### ADDRESS,

BY I. J. WETHERBEE, D. D. S.

Read before the Massachusetts Dental Society, at its Second Annaal Meeting, May 24, 1866.

*Mr. President, and Gentlemen of the Massachusetts Dental Society:*

In entering upon the third year of our existence, it seems to me highly important that we retrospect the past; that we may gather instruction from events which have transpired, and which necessarily form a part of the history of our young and prosperous Society.

An all-wise Providence has dealt tenderly with us, for it is not said of any member of this numerous circle of professionally devoted men, "The silver cord is loosed, or the golden bowl broken, or the pitcher broken at the fountain, or the wheel broken at the cistern."

All we are, and ever hope to be, is due to the inspiring truths of revelation and science. There is no record of higher authority than the sacred volume, wherein is recorded, "Whatsoever a man soweth that shall he also reap," and which also declares, "The hand of the diligent maketh rich." Whatever degree of knowledge and proficiency we may have attained is due to an appreciation of these truths, and a personal, patient application of the talents which God has given us for good and practical purposes.

Utopian *theorizers* may stir up dust, to the disadvantage of their fellows, and becloud the minds of the less observing for the time being: but truth is mighty and must prevail; for "It seeketh the light;" indeed, it bringeth to light things that were hidden. *To know the truth*, and to walk in the *light* thereof, and to be "living epistles known and read of all men," I consider to be no less our duty, as members of an active society, than it is for the moralist to present a blameless record of himself to his fellow men.

Dental Surgery, our chosen specialty, embraces vast truths which are accessible only as we remove superficial incrustations which environ them, and by careful analysis separate the *true* from the *false*, so fully as to be able to recognize their adaptation and application to the highest claims of man.

During the year, the meetings of the Society have been well attended, which is commendable for the past, and augurs well for the future; very general harmony has characterized our deliberations; a variety of topics have been discussed, intimately related to our specialty, which have produced in some minds a thorough conviction that *too little* has been known of the best modes of practice in years gone by, and aroused a commendable zeal to perfect themselves in all that appertains to a skillful practitioner of dental surgery.

We have also learned that in every man there are valuable and useful experiences, which only need the light of social intercourse to command our respect, and sometimes our admiration. *Professional reticence*, which involves exclusiveness, has had its day of doubtful glory, and must be classified with the greed of the miser who hoards his gold for his own pleasure; whose heart is encased as with steel, and answereth not the calls of the needy, however great their extremity. To be permeated with this truth, "It is more blessed to give than to receive," is to become resolved to that *status* which makes a man useful with reference not so much to "dollars and cents" as to the good he may do.

The confession of several members of this Society that they have been materially or practically benefitted by clinics and various discussions, is the best argument I can offer in favor of the practical utility of organized effort, and the elucidation of the statement previously made. This benefit does not accrue solely to the advantage of the practitioner of dental surgery, but may be felt and duly appreciated by his patients, in the greater thoroughness and comfort obtained from operations performed.

The ordeal of *dental operations* to very many patients is a very trying one; sometimes so much so that they prefer to lose valuable members of the *dental circle*, rather than submit to the infliction of pain, unavoidable in obtaining favorable results. The acquisition of knowledge which shall leave this *incarnate demon* shorn of his power to augment the miseries of a professional hour is an attainment realized by comparatively few, and by them only as they have labored to become masters of the varied conditions and phases which are continually presenting themselves for their consideration.

It is not to be expected, however desirable it might be, that we shall see eye to eye in all matters pertaining to the practice of dental surgery. Previous education, or the want of it, have established opinions in the minds of many, which have grown with their years of practice, until a confirmed habit of thought limits the proper exercise of judgment, and naturally forestalls advancement into fields of rich discoveries, in the gleaning of which great possessions of power would be obtained.

Whatever may be the diversity of opinion with reference to this or that theory, one kind of practice or another, we should, by a suggestive style of debate, show at least that there is "unity in diversity," which is beautifully illustrated in the harmony of nature. Schismatic amplifications afford no proper nourishment to a body corporate, but the reverse; extending their miasmatic contagion to other unaffected parts. By proper care the *doubtful* may be eliminated from the *positive*, without hurt to the body where such antagonisms have unfortunately existed.

From various stand-points men have constructed their theories, and have practiced them with greater or lesser success, according to their correctness. Idiomatic precision of statement does not necessarily make a proficient in the highest order of dental practice. Theories elaborated into a profuse generalization lose much of their force for want of centralization. Ambiguity of statement carries us away from the heart of the subject in question, shutting out the light and beauty which always appear when truth is sought for with an humble, patient mind.

Classifications and distinctions are worthless unless clothed with true merit. No ephemeral display will suffice to carry forward the work to be done. It can only succeed in the hands of earnest workers, who shall persistently, by precept and example, set forth and impress the minds of others with those inspiring truths which awaken to life and activity the slumbering abilities that lie scattered around the golden fields of promise. Facts are weighty when aggregated from those varied experiences which are peculiar to the personal history of every individual worker in this department of science.

The history of the dental profession (which is the oldest of specialties), in its rise and progress, is not unlike other professions, for it has had its day of small things, in character, ability, and success. We have but to refer to the historic repute of surgery in Europe, when it first took up the scalpel, to recognize a striking similarity. Being universally subject to the dictation of the medical profession, it was without character. And whatever of ability might be displayed, or success obtained, he was "only a surgeon."

But how changed! For many years the professional surgeon has held a rank second to none among all the liberal professions, either in Europe or America. But how has this change transpired? Certainly not because human life is more valuable now than formerly, or because an advancement in intelligence enables us to award a larger meed of praise to him who dexterously wields the scalpel. Surgery has been made a specialty; herein is the problem of its success solved. And this demonstrates how fully a branch of science may become invested with power, when talent is appropriated or applied to a specific end.

Knowledge is power, however gained, but there are certain channels through which knowledge may be systematically obtained. I would therefore urge upon the consideration of every young man who contemplates entering upon the study of dentistry the practicability of first graduating at some Medical College, and of attending a full course of instruction at some one of the Dental Colleges; that he may be thoroughly furnished unto every good work which appertains to his specialty. If he cannot graduate at more than one for want of means to pay his way, let the Dental College have by far the greater preference.

I cannot too strongly urge the necessity of the latter course; for the time has come when every student of dentistry should feel the importance of establishing a public confidence in his ability to meet, in a scientific manner, the exigencies of every case which may come under his professional notice. It is due both to himself and patients that all proper assurances and guaranties be at hand, that the line of demarcation may be so obvious, that *empirics* and *charlatans* will find their occupation gone.

Dental Colleges, of which there are five, have played an important part in redeeming the profession from the condition of a mere handicraft, and have given to the world many intelligent men, whose ability and scientific attainments claim for them honorable mention and high regard.

The formation of Dental Societies is another means of promoting the welfare of the profession. Every practical dentist should seek for associate membership, and rest not until he obtains it. He may have to work hard and apply himself to diligent study before the goal is reached; but all s will accrue to his personal benefit.

It is true that in some localities *Mutual Admiration Societies* have kept men aloof from dental organizations, as though they feared contamination from professional contact. It is useless, however, to oppose the progressive tendencies of the times. We therefore say to *old fogies*, or to the *pseudo conservative* men, to beware of their opposition to concentrated effort, lest YOUNG AMERICA should overwhelm them by discovering to them the darkness of their own conceit.

The time has passed when with any show of justice it could be reasonably said of the qualified dental practitioner, "He is only a dentist." We have become a positive necessity to every household; moreover, our profession has so far advanced, through careful study and patient labor, that the results are sure of subserving the positive wants of our patients; and to be without a dentist is the exception and not the rule.

No intelligent man or woman will hold a thoroughly qualified dental surgeon second to the medical practitioner, only so far as dentistry is a specialty, and therefore bears a limited relation to the whole of medical practice. From what depths of woe and languishing estate are patients brought to enjoy freedom from pain, and "how removed are they from the appearance of premature old age." How many have been recovered of numerous ills which have baffled the skill of the medical practitioner, by setting their "dental apparatus" in order, when, as by magic, "health came again."

To practical dentists the world is indebted for the most valuable treatises or works on the human teeth and their diseases. Almost every graduate from a Medical College can say with truthfulness, "I was only instructed in the *order of development of both first and second dentition.*" In fact, the text-books used in Medical Colleges and Schools only treat of the teeth with reference to their number and the order of their eruption.

The rapid advance of dentistry as a specialty, holding, as it does, such intimate relation to medical practice, claims honorable acknowledgment by the liberal professions of to-day. If they are slow in granting a formal recognition, they are swift to avail themselves of the most distinguished services that can be obtained.

As to the demand for and the propriety of establishing a Dental College in the City of Boston, for the accommodation of students of the New England States and the British Provinces, there is at present a diversity of opinion. That such an enterprise would be attended with desirable results, provided it were commenced and carried through with untiring energy, no one will doubt. But harrassing fears of failure seem to hide from view the possibility of accomplishing so great a work.

Are there not men of wealth in this community who will gladly aid us in establishing, on a firm basis, a school of instruction, where young men may be fitted for all the practical duties belonging to our profession? Until an effort is made in this direction, we shall never know how well or how poorly we might have succeeded. I trust the subject will receive from our hands that consideration which it so eminently deserves.

It is quite evident that there is yet much to be done, in private as well as associate capacity, in reforming abuses which have long been tolerated by dental practitioners and the public at large. The low fees charged by many for dental operations are a shame and disgrace to the profession. But, at the same time, it is all and more than they are worth; and when such are expostulated with, in reference to the quality of their operations and the fees charged, their reply is, "My patients will not pay me for good, first-class operations."

Now one of two things is true: either they cannot perform good and substantial operations required by patients generally, or they are a party to a system of empiricism which every honorable man should scout, from the early rising of the sun to the going down thereof.

Are the public to be the measurers and weighers of professional excellence, and wrest from individual practitioners the right and privilege to be honest, upright men? or shall they dictate to us a programme alike discreditable to them and to us? Verily, nay! But what shall be done when the patient has not the means wherewith to pay for thorough operations? Do your work well and thoroughly, if not half paid for it. You will then have the satisfaction, at least, of not having sacrificed your reputation for dollars and cents; nay, more, and the comfort of your patients, which is not to be computed by any monied consideration. The practice alluded to is a pernicious one; for it keeps at a low standard many whom we believe to be honest, but mistaken men, thereby entailing injury and disgrace upon the profession.

Let every man so avail himself of the facilities now easy of access as to acquit himself at the bar of his own enlightened conscience, and he will have little difficulty, if he is competent, in providing bread for himself and family, and honoring in some good degree the profession of which he is ever so humble a member.

Another evil exists, which calls for united action for its removal. I refer to the practice among some dentists (not of this Society, however,) of turning out *yearlings, or unfledged dentists*, who go forth with the prefix of *doctor* to their name, without any right to the assumption of such a distinguishing title, other than that they have entered in at one door, and passed out of another, of some dental office or laboratory; the head of which is first and mostly culpable in setting in motion this base imposition upon the community. I hold that three years of diligent study and faithful practice, with the promise of graduating at some one of the Dental Colleges, is the only honorable way of rendering unto the profession its due, and affording the public those professional safeguards which they have a right to claim at our hands. Let it be understood that no man can have access to our offices as a student unless he shall comply with the above regulations, and a severe scourge to suffering humanity will be abated.

I will also call your attention to the practicability of frequently holding clinics, as being one of the best means of demonstrating "*the best modes of practice*" in the operative department of our profession. Light may be so arranged at clinical demonstrations as to allow of convenience in operating on many teeth which may come under our notice.

In conclusion, let a goodly fellowship unite us in the bonds of professional esteem, that we may jointly prosecute the work before us, with a view to the advancement in knowledge and skill commensurate with the greatness of our profession, and the increasing demand for our services. May our intelligence, skill and uprightness be worthy of emulation; that we may provoke others to eminent good works in the field of our chosen labor.

It is our duty to transmit to those who shall come after us, not only an honorable record as men, but to make that record replete with evidences of professional excellence, which shall command the respect and admiration of all true lovers of science. It needs no prophetic vision to measure the future, to foretell the rapid advancement of our profession in intelligence, ability and character. Its rapid growth for the past fifteen years is the earnest of this expectation; and its fulfillment the younger members of this Society may live to see accomplished. And may we never forget that

"Years following years steal something every day;
At last they steal us from ourselves away."

